# Comprehensive Analysis of *Phaseolus vulgaris SnRK* Gene Family and Their Expression during Rhizobial and Mycorrhizal Symbiosis

**DOI:** 10.3390/genes13112107

**Published:** 2022-11-13

**Authors:** Carolina Cervera-Torres, Manoj-Kumar Arthikala, Miguel Lara, Lourdes Blanco, Kalpana Nanjareddy

**Affiliations:** 1 Ciencias Agrogenómicas, Escuela Nacional de Estudios Superiores Unidad León-Universidad Nacional Autónoma de México (UNAM), Guanajuato C.P. 37689, Mexico; 2 Departamento de Biología Molecular de Plantas, Instituto de Biotecnología, UNAM, Cuernavaca C.P. 62210, Mexico

**Keywords:** genome-wide analysis, SnRK, *Phaseolus vulgaris*, *Rhizobium*, arbuscular mycorrhizae, protein interactions

## Abstract

*Sucrose non-fermentation-related protein kinase 1* (*SnRK1*) a Ser/Thr protein kinase, is known to play a crucial role in plants during biotic and abiotic stress responses by activating protein phosphorylation pathways. *SnRK1* and some members of the plant-specific *SnRK2* and *SnRK3* sub-families have been studied in different plant species. However, a comprehensive study of the *SnRK* gene family in *Phaseolus vulgaris* is not available. Symbiotic associations of *P. vulgaris* with *Rhizobium* and/or mycorrhizae are crucial for the growth and productivity of the crop. In the present study, we identified *PvSnRK* genes and analysed their expression in response to the presence of the symbiont. A total of 42 *PvSnRK* genes were identified in *P. vulgaris* and annotated by comparing their sequence homology to *Arabidopsis SnRK* genes. Phylogenetic analysis classified the three sub-families into individual clades, and *PvSnRK3* was subdivided into two groups. Chromosome localization analysis showed an uneven distribution of *PvSnRK* genes on 10 of the 11 chromosomes. Gene structural analysis revealed great variation in intron number in the *PvSnRK3* sub-family, and motif composition is specific and highly conserved in each sub-family of *PvSnRK*s. Analysis of *cis*-acting elements suggested that *PvSnRK* genes respond to hormones, symbiosis and other abiotic stresses. Furthermore, expression data from databases and transcriptomic analyses revealed differential expression patterns for *PvSnRK* genes under symbiotic conditions. Finally, an in situ gene interaction network of the *PvSnRK* gene family with symbiosis-related genes showed direct and indirect interactions. Taken together, the present study contributes fundamental information for a better understanding of the role of the *PvSnRK* gene family not only in symbiosis but also in other biotic and abiotic interactions in *P. vulgaris*.

## 1. Introduction

Sucrose nonfermenting 1 (SNF-1)-related protein kinase (SnRK1), animal homologue AMP-activated protein kinase (AMPK) and yeast homologue Sucrose Non-Fermenting1 kinase/plant SNF1-related kinase1 (SNF1) are highly conserved serine/threonine protein kinases that function as cellular energy sensors activating catabolism and repress anabolic processes [1]. These protein kinases function as heterotrimeric complexes, with α, β and γ subunits carrying out catalytic and regulatory functions [2,3,4]. All SnRK proteins have a conserved N-terminal kinase domain and variable regulatory C-terminal domain, which mediates interaction between the β and γ subunits [1]. The prerequisite for AMPK/SNF1/SnRK1 functioning as kinases is phosphorylation of the T-loop at the N-terminal kinase domain [5,6,7].

*SnRK*s in plants are classified into *SnRK1*, *SnRK2* and *SnRK3* sub-families based on sequence similarity and structural organization [1,8]. *SnRK1* is the homologue of *AMPK*, and *SNF1*, *SnRK2* and *SnRK3* are unique to plants and evolved from the *SnRK1* family via gene duplication during plant evolution, and play a key role in the stress, calcium and ABA signalling pathways with epigenetic and metabolic responses [8].

The *SnRK1* sub-family consists of an N-terminal kinase domain, a ubiquitin-associated (UBA) domain and a C-terminal kinase-associated 1 (KA1) domain [7]. The *SnRK1* gene family is a comparatively smaller sub-family primarily found to be core sensors of energy deficit in plants. The mode of regulation is found to be through downstream phosphorylation and inhibition of the enzymes HMG-CoA reductase (HMGR), sucrose phosphate synthase (SPS), nitrate reductase (NR) and seaweed phosphate synthase 5 (TPS5) [9,10,11] involved in metabolic regulation and plant developmental processes. *SnRK1* genes are pivotal in various signalling pathways, such as meristem development, cell cycle regulation and pathogen responses [12].

The *SnRK2* sub-family harbours the regulatory C-terminal domain containing acidic amino acids, either Glu or Asp [7]. The regulatory role of the *SnRK2* sub-family involves activation of basic region-leucine zipper (bZIP) transcription factors in association with an epigenetic mechanism [13,14]. The *SnRK2* sub-family has been well studied in plants for its involvement in abiotic stress, either ABA mediated or ABA independent. The *SnRK2* sub-family in *Arabidopsis* is classified into three groups: ABA dependent, drought responsive and ABA activated [15,16]. In *Arabidopsis* and rice, the *SnRK2* sub-family is larger than that of *SnRK1*, with 10 members found to be abiotic stress responsive and activated by ABA and to phosphorylate transcription factors of the ABA-response element-binding protein class (AREBP) [17,18,19,20,21].

The *SnRK3* sub-family, also named *CIPK*s (CBL-interacting protein kinases), contains two conserved domains at the C-terminus, including NAF (named with conserved amino acids N, A and F) and PPI (protein–protein interacting) domains [1]. The *SnRK3* sub-family is the largest in all plants studied thus far; these proteins transport Na^+^, K^+^ and NO_3_ ions, along with their binding ability to Ca^2+^-dependent CBL to regulate downstream genes and enhance abiotic stress tolerance [22,23,24]. SnRK3 responds to abiotic stress factors such as salinity stress, as reported in rice, Arabidopsis, and tomato [25,26,27]. The other important mechanism of the SOS (salt overly sensitive) system is provided by SOS2/*At*CIPK24 (salt overly sensitive 2), which is a member of the *SnRK3* sub-family in *Arabidopsis thaliana*. As a Na^+^/H^+^ antiporter, it improves plant salt tolerance by maintaining ionic homeostasis [28,29]. Taken together, *SnRK*s represent one of the larger gene families in plants involved in a variety of regulatory mechanisms including metabolism, growth, development, and abiotic stress response.

Due to the varied regulatory roles played by *SnRK* genes, the recent years have seen extensive genome-wide analysis in various plant species. Starting form *Arabidopsis* [1] to economically important crops such as *Brassica napus* [30], *Cucumis sativus* [31], *Eucalyptus grandis* [32], *Fragaria ananassa* [33], *Triticum aestivum* [34], *Hordeum vulgare* [35], *Oryza sativa* [17] and *Brachypodium distachyon* [36]. Each of these analyses has revealed similar classification of the *SnRK* gene family and the highly conserved nature of gene structure and function. Curiously, these studies do not involve any legumes.

Among flowering plants, legumes are the most important agricultural crops after cereals due to their high economic value. Legumes play a central role at the food system level, both for human and animal consumption, as a source of plant proteins [37]. Furthermore, at the cropping system level, legumes are used as diversification crops in agroecosystems to break the cycles of pests and diseases and contribute to balancing the deficit in plant protein production; also at the cropping system level, legumes contribute to low-input cropping systems due to their ability to fix atmospheric nitrogen [38,39,40,41]. Legumes are distinct in their ability to establish a symbiotic interaction between nitrogen-fixing rhizobia and nutrient-transporter mycorrhizae. Although legumes are of such high economic value, some, such as *P. vulgaris*, are not considered subjects in the exploration of gene families.

A better understanding of the signalling pathways that affect the productivity and sustainability of such crop systems is particularly important. A gene family such as *SnRK* with diverse regulatory mechanisms might play a pivotal role in biotic and abiotic interactions of legumes, and greater knowledge of these aspects may help in crop improvement. In the present study, we sought to identify and understand the diversity of the *SnRK* gene family in *P. vulgaris* and elucidate their differential expression patterns under rhizobial and mycorrhizal symbiotic conditions.

## 2. Materials and Methods

### 2.1. Identification and Alignment of PvSnRK Family Genes in P. vulgaris

The amino acid and nucleotide sequences of the *A. thaliana SnRK* gene family were downloaded from NCBI (https://www.ncbi.nlm.nih.gov/, accessed on 14 July 2021). Homologues of *AtSnRK*s were searched in the Phytozome (https://phytozome.jgi.doe.gov, accessed on 14 July 2021) and Legume Information System (https://legumeinfo.org, accessed on 25 July 2021) databases using BLASTN and BLASTP methods. The respective nucleic acid and peptide sequences were downloaded from the online tool PhytoMine from the plant comparative genomics portal Phytozome v12.1 for further analysis and annotation. The hidden Markov model (HMM) and BLASTP program were applied for preliminary identification of *PvSnRK* proteins. Local BLASTP (E-value-20) searches were performed based on hidden Markov model (HMM) profiles of *PvSnRK* gene domains from the Pfam database (http://pfam.janelia.org/, accessed on 18 August 2021). The SMART database (http://smart.embl-heidelberg.de/, accessed on 18 August 2021) was used to confirm *PvSnRK* gene sequences [42], as were the Pfam database [43] and the NCBI Conserved Domain database [44].

MEME and motif discovery tools were employed to filter out *PvSnRK* homologues based on domain structure [45]. The chromosomal localization of *PvSnRK* gene family members was verified in the Phytozome v12.1 database, chromosomal images were drawn using the EnsemblPlant tool [46], and the scale was determined based on Wang et al. (2016) [47]. The number of amino acids, molecular weights (MWs) and isoelectric points (pIs) of *PvSnRK* proteins were calculated using tools from ExPASy (http://www.expasy.ch/tools/pi_tool.html, accessed on 2 September 2021). Subcellular localization of *PvSnRK* proteins were predicted by ProtCompv.9.0 (http://linux1.softberry.com/berry.phtml, accessed on 10 September 2021), BaCelLo (http://gpcr.biocomp.unibo.it/bacello/pred.htm, accessed on 10 September 2021) [48], LocTree3 (https://rostlab.org/services/loctree3/, accessed on 10 September 2021) [49], WoLF PSORT (https://wolfpsort.hgc.jp/, accessed on 10 September 2021) [50] and Cello (http://cello.life.nctu.edu.tw/, accessed on 10 September 2021) [51].

### 2.2. Phylogenetic Analysis of PvSnRK Family Genes

Multiple sequence alignment of *PvSnRK*s and *AtSnRK*s was performed using ClustalW. Based on the alignments, phylogenetic analysis of the aligned sequences was carried out using Molecular Evolutionary Genetics Analysis (MEGA XI) with the neighbour-joining (NJ) method and the JTT + I + G substitution model with 1000 bootstrap replicates and default parameters [52].

Conserved motifs in the *PvSnRK* gene family in *P. vulgaris* were identified using the Multiple Expectation Maximization for Motif Elicitation (MEME) online program (http://meme.sdsc.edu/meme/itro.html, accessed on 2 October 2021) with the following parameters: number of repetitions = any, maximum number of motifs = 20; and optimum motif length = 6 to 100 residues. The gene structure of the *PvSnRK* gene family was analysed using the Gene Structure Display Server online program (GSDS: http://gsds.cbi.pku.edu.ch, accessed on 10 October 2021) [53].

Sequences 2 kb upstream of *PvSnRK*s were downloaded from the Phytozome database. The plant transcriptional regulatory map (http://plantregmap.gao-lab.org/, accessed on 25 October 2021) was used to analyse the promoter sequences.

### 2.3. Calculation of Ka/Ks and Dating of Duplication Events

To identify putative orthologues between two different species (A and B), each sequence from species A was searched against all sequences from species B using BLASTN; each sequence from species B was also searched against all sequences from species A. The two sequences were defined as orthologues when reciprocal best hits were each within ≥300 bp of the two aligned sequences. The duplication period (Million Years ago, MYA) and divergence of each *PvSnRK* gene were calculated using the following formula: T = Ks/2λ (λ = 6.56 × 10^−9^) [54]. The calculation of Ka and Ks was performed using TBtools. Graphs were developed with R studio with the R commander package [55].

### 2.4. Protein Interaction Network and Gene Ontology Analysis

A high-expression candidate gene protein interaction network was analysed using STRING v11.0 (https://stringpreview. org/, accessed on 15 March 2022) and visualized using Cytoscape v3.9.1 (http://www.cytoscape.org/download.php, accessed on 21 March 2022). Finally, enrichment analysis of Gene Ontology (GO) terms was performed using the online tool AgriGO (v2.0) (http://systemsbiology.cau.edu.cn/agriGOv2/, accessed on 6 April 2022) [56].

### 2.5. Transcriptome Profiling and RT–qPCR Analysis

Data on differential expression of *SnRK* genes in *P. vulgaris* tissues under nitrogen treatments and after inoculation with *Rhizobium tropici* (CIAT899) were obtained from the PvGEA website (https://plantgrn.noble.org/PvGEA/, accessed on 12 January 2022). Previously, we performed global transcriptome profiling in *P. vulgaris* L. cv. Negro Jamapa roots colonized by *Rhizophagus irregularis* spores or *Rhizobium tropici* strain CIAT899 [57]. The present study uses the same transcriptomic data to obtain expression profiles of *PvSnRK* family genes under both types of symbiotic conditions. Heatmaps were constructed with fold-change values applying the R package (https://www.r-project.org/, accessed on 14 January 2022).

To validate the RNA-seq data, we surface-sterilized *P. vulgaris* L. cv. Negro Jamapa seeds and germinated them as described by Nanjareddy et al. [57]. Two-day-old germinated seedlings were transplanted into sterile vermiculite and inoculated with *R. irregularis* or *R. tropici* according to Nanjareddy et al. [58]. Total RNA preparation and RT–qPCR analysis were carried out according to Quezada et al., 2019 [59].

## 3. Results

### 3.1. Identification of PvSnRK Protein Orthologues in P. vulgaris

A BLAST search was carried out using *Arabidopsis SnRK*s as a reference, and a total of 42 genes were identified based on conserved domains in each sub-family (Table S1). The sub-family *PvSnRK1* was identified by the domain KA1 (PF02149), the *PvSnRK2* sub-family by the OST domain [60], and the *PvSnRK3* sub-family by the NAF/FISL domain (PF02149). The genes were named *PvSnRK1.1*, *PvSnRK1.2*, *PvSnRK2.1*-*PvSnRK2.11* and *PvSnRK3.1*-*PvSnRK3.29* based on sequence homology with the *Arabidopsis SnRK* family. The amino acid length of the 42 *PvSnRK* gene family members ranged from 310 aa (*PvSnRK2.11*) to 528 aa (*PvSnRK1.2*), corresponding to molecular weights of 60.54 to 35.67 kDa (Table 1).

The theoretical isoelectric point of *PvSnRKs* (PI) ranged from 4.7 to 9.24, with *PvSnRK1* sub-family members showing a basic PI, the *PvSnRK2* sub-family being mostly acidic (4.7–6.65) and the *PvSnRK3* sub-family being slightly acidic to highly basic (6.4–9.37). Subcellular localization analysis was carried out using ProtComp v.9.0, and the results showed *PvSnRK1s* to localize to the extracellular space; *PvSnRK2s* mostly localized to the nucleus and plasma membrane and *PvSnRK3* sub-family members to the plasma membrane (Table 1). However, the subcellular localization analysis through different software showed some variation, as depicted in Table S3.

### 3.2. Phylogenetic and Structural Analyses

**Figure 1:**
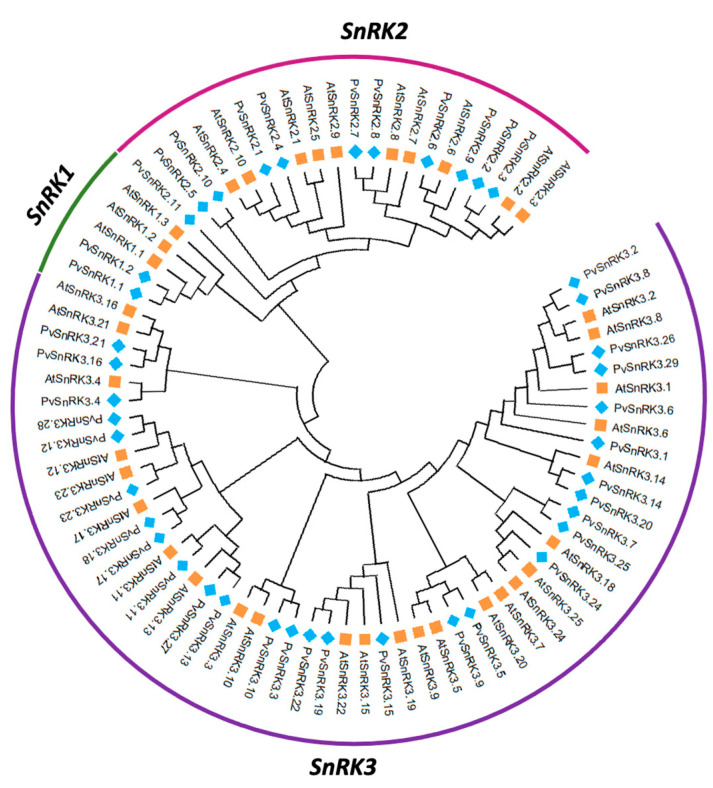
Phylogeny of the *SnRK* gene family in *Arabidopsis thaliana* and *Phaseolus vulgaris*. Phylogenetic tree constructed with distance and neighbour-joining method using deduced full-length protein sequences of *SnRK* family genes of two species. The phylogenetic tree was constructed using MEGA XI software with the neighbour-joining tree method with 1000 bootstrap values.

To determine the evolutionary relationship among *Arabidopsis* and *Phaseolus SnRK* superfamily genes, a phylogenetic tree was constructed using the protein sequences of 42 *PvSnRK* genes and 34 *AtSnRK* genes using the neighbour-joining (NJ) method with 1000 bootstrap replications (Figure 1, Figure S2). The accession numbers or locus IDs of the *SnRK* genes are listed in Table 1 and Supplementary Table S2. The resulting tree categorized the *PvSnRKs* and *AtSnRKs* into four clades, indicating that the ancestral genes of these two clades diverged before Brassicaceae and Fabaceae separated. In the *P. vulgaris* phylogeny, clade I contains *PvSnRK2* sub-family members represented by the OST domain, clade II contains the *PvSnRK1* sub-family containing the KA1 domain, and clades III and IV contain the NAF/FISL domain and 3 and 26 members of the *PvSnRK3* sub-family, respectively (Figure S1). The combined phylogenetic analysis of *Phaseolus* and *Arabidopsis SnRK* proteins also showed a similar distribution, whereby the proteins from two species appear scattered across the branches of the evolutionary tree, suggesting that they experienced duplications after the lineages diverged.

**Figure 2:**
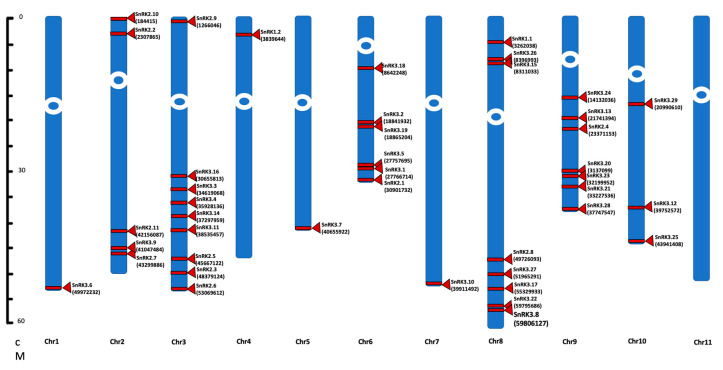
Chromosomal localization of the *SnRK* genes. The sequences of 42 genes of *SnRK* genes were identified on *P. vulgaris* genome. The chromosomes are represented by the numerically distributed blue bars. Red bands and triangles indicate the location of each gene on the chromosome. The numbers in the parenthesis represent the start site of the individual *SnRK* gene location on the *P. vulgaris* genome.

To examine the evolution of *Phaseolus SnRK* genes, their chromosomal distribution was determined. *PvSnRK* superfamily genes are distributed across 10 pairs of homologous chromosomes among 11 pairs in the *P. vulgaris* genome. *PvSnRK1* genes are located on chromosomes 4 and 8 and *PvSnRK2* genes on chromosomes 2, 3, 6 and 8. *PvSnRK3*s are located on nine chromosomes, where chromosomes 1 and 11 are the exceptions, and chromosome 3 contains the highest number of *PvSnRK* genes (nine *PvSnRK*s), followed by chromosome 8 (eight *PvSnRK*s). Chromosomes 1, 4, 5 and 7 each have one gene each, as shown in Figure 2 and Table 1.

**Figure 3:**
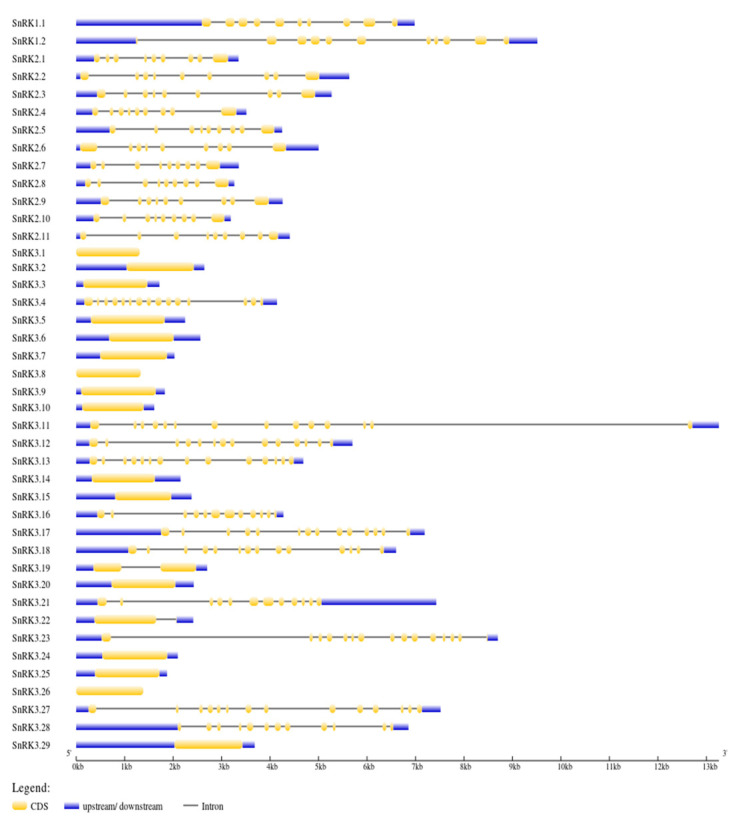
The exon–intron organization of the corresponding *SnRK* genes. The exons are represented by the yellow boxes, introns by black lines, and the untranslated regions (UTRs) are indicated by the blue boxes. The sizes of the exons and introns can be estimated using the scale detailed at the bottom.

Intron–exon analysis was carried out to obtain better insight into the structure of PvSnRK genes. PvSnRK gene family members exhibited a great variation, from 1 to 14 introns, as shown in Figure 3 and Table 1. In the PvSnRK1 sub-family, PvSnRK1.1 and PvSnRK1.2 have 9 and 10 introns, respectively, and all members of the PvSnRK2 sub-family have 8 introns each. A great variety in introns numbers was found in the sub-family PvSnRK3, with 16 PvSnRK3 members having no introns, PvSnRK3.19 having 1 intron, PvSnRK3.21 and 3.28 having 11 introns, and PvSnRK3.4 having 14 introns; the remaining eight PvSnRK3 sub-family members have 13 introns. This divergence in introns numbers indicates that exon gain, and loss occurred during evolution of the PvSnRK gene family. These findings are corroborated by the clades in the phylogenetic analysis, where clade I contains all PvSnRK2 members, clade II has PvSnRK1 individuals, and PvSNRK3 members are divided into clade III, with genes comprising 11 and 14 introns. Finally, clade IV includes all the remaining members of PvSNRK3 (Figure S1).

**Figure 4:**
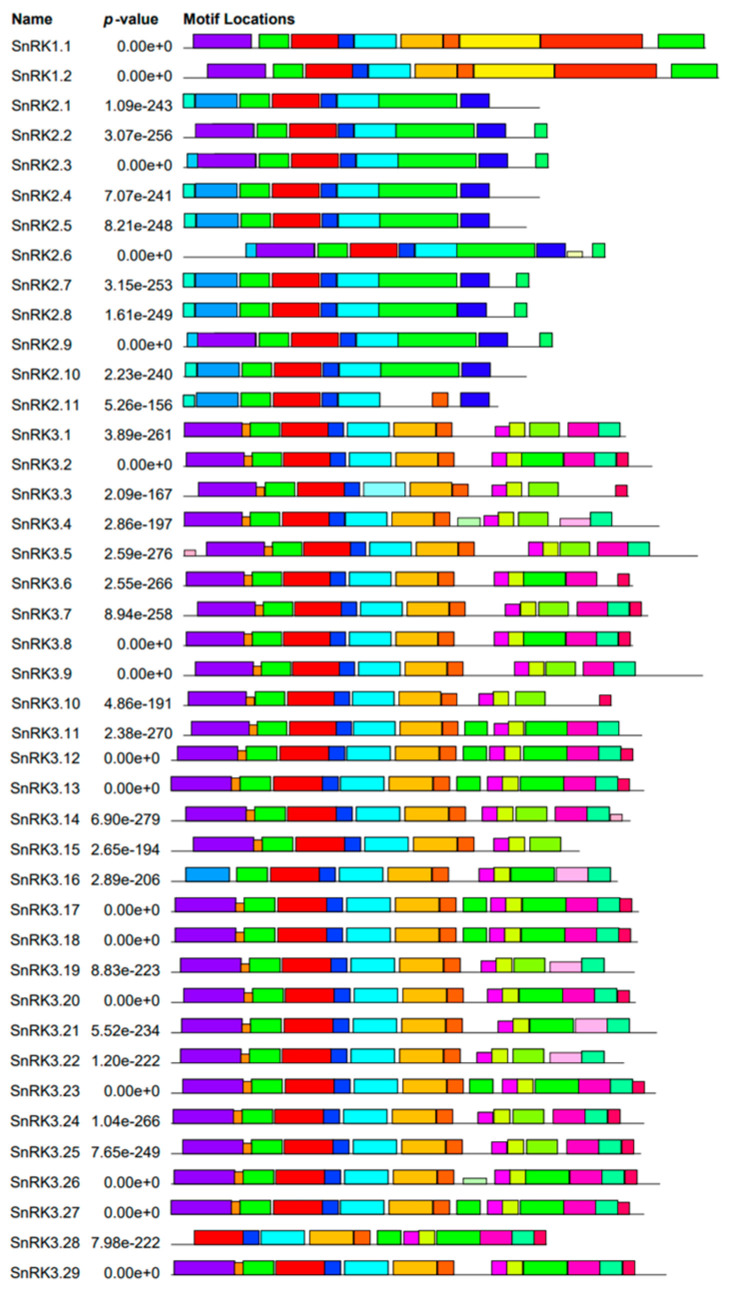
Schematic representation of putative conserved motifs identified MEME in *Phaseolus* SnRK proteins. Putative conserved motifs shared by *Phaseolus* SnRK proteins were mined in MEME program. Twenty-five motifs are indicated by differently coloured boxes and the regular motif sequences are shown in the Table S4.

A search for conserved motifs in all 42 *PvSnRK* proteins using the MEME program revealed a total of 25 conserved motifs, named from 1 to 25. The identified motifs were annotated in Pfam, and the details of the putative motifs are shown in Table S4. Motifs 1-4 are designated protein kinase domains and are found in all three sub-families. Motif 8, a kinase domain associated with kinase1 and KA1, and motifs 10, 11, and 20, which encode an NAF domain, are only present in *PvSnRK3*. Ubiquitin-associated domain 20 was found in *PvSnRK1* and *PvSnRK3*. Furthermore, motifs 5 and 13, designated protein superfamily kinase domains, were found only in the *PvSnRK1* and *PvSnRK3* sub-families (Figure 4). The remaining motifs were not annotated functionally.

### 3.3. Ka/Ks and Gene Duplication

To further explore evolutionary constraints on *Phaseolus PvSnRK* genes, synonymous (Ks) and nonsynonymous (Ka) substitutions per site and their ratio (Ka/Ks) and divergence time of paralogous and orthologous *SnRK* family genes were calculated for *AtSnRK* orthologues of *PvSnRK*s (Table 2 and Table S5). The Ka/Ks ratio among all *SnRK* sequences was lower than 1, indicating purifying selection. These Ka/Ks ratios suggest the conservation of *SnRK* homologues in terms of both sequence and biological function [61].

### 3.4. Cis-Elements in Promoter Regions of PvSnRKs

**Figure 5:**
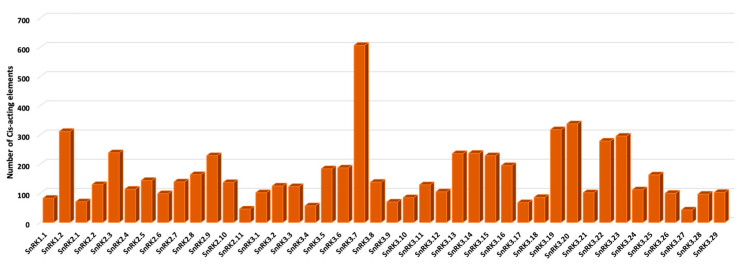
Frequency of *PvSnRK cis-*acting elements in *P. vulgaris*.

To determine the gene expression pattern of *PvSnRK*s, the 2 kb region upstream of the CDS was analysed using the PlantRegMap database. Among all transcription factors recorded, ERF and MYB were found to be the most abundant. *PvSnRK3.7* contained the greatest number of *cis*-elements (607) in the examined regulatory region, with 286 ERF binding sites and 49 MYB and 44 C2H2 sites. *PvSnRK 3.20* has 338 TF sites; this was followed by *PvSnRK 3.19* with 318 TFs, with 111 TFs being ERF TFs and 56 being bHLH TFs, and *PvSnRK 1.2*, with 312 TFs, with 100 being NAC TFs, 45 being MYB TFs and 40 being ERF TFs (Figure 5, Table S6, Figure S3). The most abundant TFs, ERF, C2H2, bHLH, NAC and MYB identified in *PvSnRK*s were also found by symbiosis related studies in other species such as *M. truncatula* and *L. japonicus* (Table 3).

MYB transcription factors promote expression of genes involved in cell proliferation and differentiation. ERFs are involved in regulation of developmental processes in response to stimuli, and NAC, C2H2 and bHLH are involved in the pathogen response, cell proliferation and development.

### 3.5. GO Analysis

**Figure 6:**
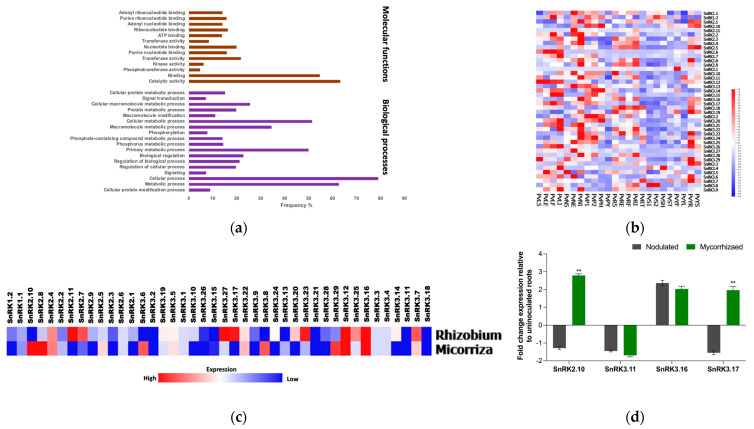
Gene Ontology and in silico expression analysis of *SnRK* genes. (**a**) Gene Ontology enrichment analysis of molecular analysis and biological processes for the members of *Phaseolus SnRK* genes. (**b**) Tissue-specific expression profiles of *Phaseolus SnRK*s. Heat map expression profiles of *SnRK* genes in various *Phaseolus* tissues. The transcriptome data across different tissues were extracted from the *P. vulgaris* gene expression atlas (*Pv*GEA). (**c**) Heat maps showing *PvSnRK* gene expression patterns specific to AM and rhizobial colonization. Colour bar shows the fold-change range, with red and blue representing upregulation and downregulation, respectively. The heat map was generated by R using the Fragments per kilobase of exon model per million reads mapped (FPKM) values of each *SnRK* gene. (**d**) RT-qPCR analysis showing relative expression of *Phaseolus SnRK2.10*, *SnRK3.11*, *SnRK3.16*, and *SnRK3.17* genes. Candidate genes were selected and corresponding transcript accumulation under mycorrhized and nodulated conditions was quantified by RT-qPCR. RT-qPCR data are the averages of three biological replicates (n > 9). The statistical significance of differences between mycorrhized and nodulated roots was determined using an unpaired two-tailed Student’s *t*-test (** *p* < 0.01). Error bars represent means ± Standard error mean (SEM).

Gene Ontology analysis of all *PvSnRK* genes showed them to be involved in biological processes and molecular functions, but not cellular components. The biological processes involved related to *PvSnRK*s are cellular signalling, phosphorylation, and metabolic processes such as protein, macromolecule, and phosphorous metabolism. Among molecular functions, the majority are associated with binding and catalytic activities, followed by transferase and nucleotide-binding functions (Figure 6a).

### 3.6. Expression Profiles of PvSnRK Genes in Different Tissues

Differential expression data for *PvSnRK* genes were obtained from PvGEA: Common Bean Gene Expression Atlas and Network Analysis (https://plantgrn.noble.org/PvGEA/, accessed on 16 November 2021). The expression patterns of all 42 *PvSnRK* members were analysed in 25 different tissues, including leaves, stems, shoots, pods, seeds, roots (inoculated and uninoculated with *Rhizobium*) and nodules, at different developmental stages and treatments (Figure 6b, Table S7). The lowest expression of all *PvSnRK* genes was found in young flower, seed and shoot tissues; at least one of the 42 genes was expressed in the remaining tissues. Among nodulation-related tissues, most of the *PvSnRK* genes showed high expression, except for the *PvSnRK1* sub-family, in whole roots separated from fix+ nodules collected at 21 days after inoculation (*PvRE*). Pre-fixing (effective) nodules collected at 5 days after inoculation (*PvN5*) showed very high expression of only *PvSnRK3.5* and *PvSnRK3.29* (Figure 6b).

### 3.7. Expression Patterns of PvSnRK Genes under Symbiotic Conditions

Additionally, differential expression patterns of *PvSnRK*s under rhizobial and mycorrhizal symbiotic conditions were analysed by transcriptomics at the early infection stages 5 dpi and 7 dpi, respectively. Most of the genes encoding *PvSnRK1*s and *PvSnRK3*s were found to be upregulated under both symbiotic conditions, though *PvSnRK2* was less induced during symbiosis with rhizobia. It was particularly interesting to find highly induced *PvSnRK1.2* in comparison to *PvSnRK1.1*, and among the P*vSnRK3* sub-families, *PvSnRK3.10* was highly induced, followed by *PvSnRK3.7*, *PvSnRK3.20*, and *PvSnRK3.22*. Under mycorrhizal inoculation conditions, *PvSnRK1*s and *PvSnRK3*s were all induced, and some *PvSnRK2* genes were also induced. In the *SnRK1* sub-family, *PvSnRK1.2* was more induced; among *PvSnRK2* genes, *PvSnRK2.6*, *PvSnRK2.7* and *PvSnRK2.11* were least induced. In the *PvSnRK3* sub-family, *PvSnRK3.1*, *PvSnRK3.2*, *PvSnRK3.3*, *PvSnRK3.4*, *PvSnRK3.5* and *PvSnRK3.6* were less induced than other members (Figure 6c,d).

### 3.8. PvSnRK Protein–Protein Interaction Network Prediction

A protein interaction network was constructed for *Pv*SnRK proteins based on orthologues in *Arabidopsis* using the STRING database with the highest bit score (0.9). While predicting interactions among the *Pv*SnRK proteins, we used experimentally proven, co-expressed and co-occurring protein interactions, revealing a total of 40 interacting proteins. The majority of them are phosphoprotein phosphatase (PPP) family proteins, PP2A (protein phosphatase PP1-α catalytic subunit), PP2CA/PP2CB (protein phosphatase 2A catalytic subunit α/β isoform), PPP2RA/PPP2RB (protein phosphatase 2A 65 kDa regulatory subunit A β isoform) and PPP1C (protein phosphatase PP1-α catalytic subunit)-type proteins. Phosphatases are proteins involved in substrate recognition, plant signalling pathways such as stress regulation, light, pathogen defence and hormonal signalling, the cell cycle, differentiation, metabolism, and plant growth.

**Figure 7:**
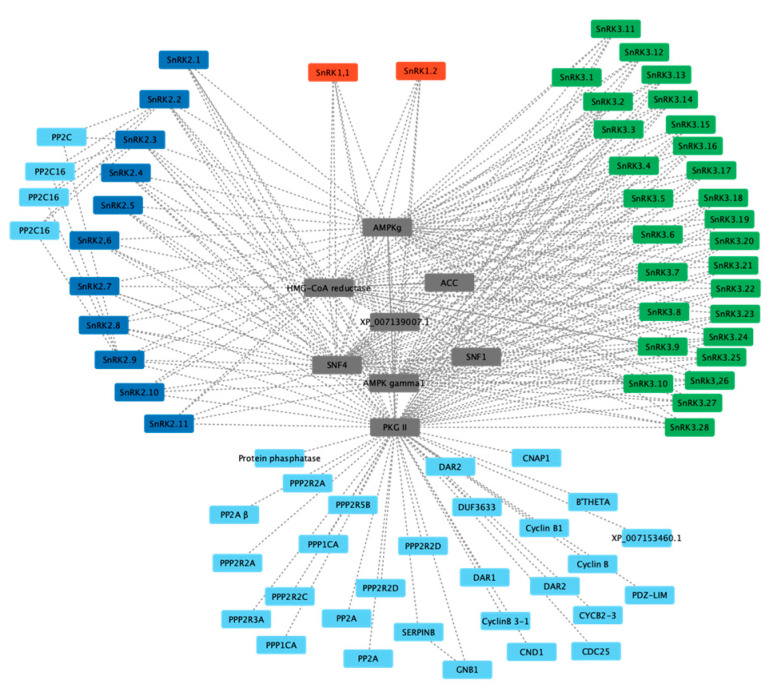
In silico prediction of protein–protein interactions among *SnRK1s*, *SnRK2s* and *SnRK3s* using the Cytoscape tool based on the Pearson correlation coefficients of the relative expression of the gene. Each node represents a protein, and each edge refers an interaction. The red-coloured box represents *SnRK1s*, blue represents *SnRK2s,* and green represents *SnRK3s*.

The next important interactions were with cell cycle proteins and cyclin B proteins. Cyclin B proteins have been implicated in plant growth and development. All *Pv*SnRK protein sub-families were found to interact with PPP family members and cyclin B proteins through PKGII, indicating their involvement in various cellular and developmental processes. SnRK2 sub-family members were found to specifically interact with PP2C proteins (Figure 7).

### 3.9. Prediction of Coregulatory and Interaction Networks of PvSnRK and Symbiotic Genes

**Figure 8:**
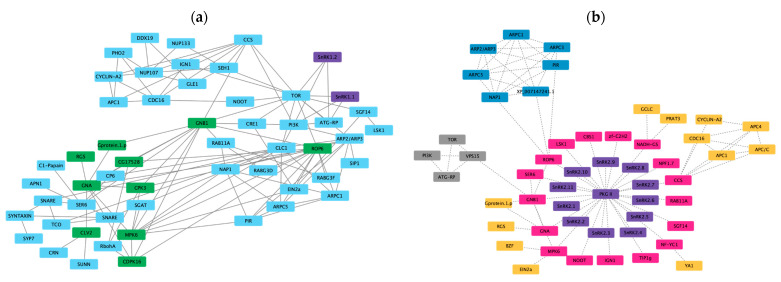
In silico prediction of protein–protein interactions among (**a**) *SnRK1s* and (**b**) *SnRK2s* with Scheme 11. *Pv*SnRK2 sub-family proteins with symbiosis-related proteins interact through 52 nodes and 81 edges. All *Pv*SnRK2 proteins interact with PKGII, which interacts with all other proteins shown in the network. Through CCS (Cell division Cycle 20 like protein) it interacts with cell cycle-related proteins such as Cyclin A2, CDC16 and APC (Anaphase-promoting complex) and through ROP6 interacted with ARP (auxin-related proteins). The prediction showed that few *Pv*SnRK2 sub-family proteins interact with symbiosis-related proteins (Figure 8b).

The symbiotic interaction between legumes and rhizobia is unique and involves a set of common symbiotic genes that regulate root nodule symbiosis and mycorrhizal symbiosis. Another 190 genes have been implicated in regulating symbiotic interactions. A total of 200 genes known to be involved in symbiosis were chosen to predict an interaction network with each of the *PvSnRK* sub-families in the STRING database. Interaction between *Pv*SnRK1 sub-family members and symbiosis-related proteins shows that *Pv*SnRK1.1 and *Pv*SnRK1.2 do not interact directly with any of the symbiosis-related genes. However, they do interact with TOR, PI3K and ATG-RP, which interact with other symbiotic genes represented by 54 nodes and 119 edges. The network mostly involves nucleoporins (NUPs), RAB proteins, auxin-responsive ARP proteins and many proteins involved in vesicle transport (Figure 8a).

**Figure 9:**
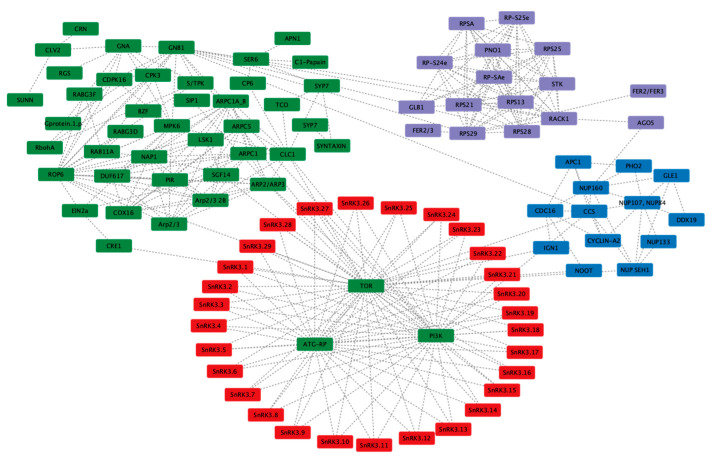
In silico prediction of protein–protein interactions among *SnRK3* subfamily proteins with symbiosis-related proteins using the Cytoscape tool based on the Pearson correlation coefficients of the relative expression of the gene. Each node represents a protein, and each edge refers an interaction. The different colours represent the different gene families.

The largest *Pv*SnRK3 sub-family, with 29 members, interacts with TOR, PI3K and ATG-RP at the first level, similar to *Pv*SnRK1 members. The predictions showed 102 nodes and 319 edges, indicating a larger network. CCS interacts with nucleoporins (NUPs), and through GNBI, they interact with several RPSs (ribosomal proteins) and a variety of symbiosis-related proteins. Network prediction showed that *Pv*SnRK3 sub-family members may be involved in regulating symbiosis in *Phaseolus* via different signalling pathways (Figure 9).

## 4. Discussion

The *SnRK* gene family, serine/threonine kinases, and its orthologues in animals and yeast are highly conserved. In plants, they have been identified as regulators of abiotic and biotic stress [80,81,82]. In recent years, genome-wide analysis of this gene family in an array of both model and economically important plant species has focused primarily on abiotic stress. *Phaseolus vulgaris* is the most important legume consumed by humans worldwide, as it is an affordable source of proteins, vitamins, minerals, antioxidants, and bioactive compounds [83]. Climate change has impacted the world’s crop yield and quality, leading to socioeconomic and food insecurity [84]. The yield quantity and quality of N2-fixing crops can be improved by several agronomic practices, such as irrigation, sowing density and *Rhizobium* application. Since common bean does not need exogenous N fertilizer application, productivity is cost effective. Any efforts towards the betterment of rhizobial association to improve N fixation in crops such as *Phaseolus* should be undertaken. The present investigation encompasses the identification, classification, and analysis of the expression patterns of the *SnRK* gene family under symbiotic conditions.

Genome-wide identification studies have been carried out, with early reports documenting the presence of various numbers of members in both monocots and dicots. A total of 39, 114, 30, 34, 26, 149, 46, 48 and 44 *SnRK* genes have been identified in *A. thaliana* [1], *Brassica napus* [30], *C. sativus* [31], *E. grandis* [32], *F. ananassa* [33], *T. aestivum* [34], *H. vulgare* [35], *O. sativa* [17] and *B. distachyon* [36], respectively. In *Phaseolus*, we identified 42 *SnRK* genes with 2 *SnRK1*s, 11 *SnRK2*s and 29 *SnRK3*s with the characteristic domains of each sub-family. As in any other species, the *SnRK2* and *SnRK3* sub-families in *Phaseolus* are larger than *SnRK1*, supporting the view that these two sub-families originated from duplication of *SnRK1* [35]. The nonmotile nature of plants exposes them to biotic and abiotic factors, and plants adopt such changes by expanding their genes and gene families. Gene and genome duplications are important events that contribute to polyploidy and genome evolution. One or multiple polyploidies are prevalent in angiosperms [85,86] and explain the large number of *SnRK2* and *SnRK3* sub-family members in all reported plant species.

In *Phaseolus*, the *SnRK* gene family is distributed on 10 of 11 chromosomes. This distribution is unlike in other species, in which all chromosomes in the genome contain at least one of these genes [1,17,30,31,32,33,34,35,36]. *Phaseolus* chr3, chr6, chr8 and chr9 show clustering of genes, mostly of the *SnRK3* sub-family.

Each of the *SnRK* sub-families has a characteristic domain; however, the N-terminal kinase domain is highly conserved across species and sub-families. Exon–intron structural diversification and motif composition play an important role in the evolution and function of many gene families [87]. *PvSnRK1* sub-family members have 10–11 exons, such as *BdSnRK1*s, *EgrSnRK1*s and *CsSnRK1*s. All *PvSnRK2*s have nine exons, similar to most *HvSnRK*s, *HcSnRK2*s, *EgrSnRK2*s, *VvSnRK2*s, *AtSnRK2*s and *BdSnRK2*s, indicating the conserved nature of these sub-families. The sub-family *PvSnRK3* can also be subdivided into exon-rich and exon-poor types, as can all other species reported thus far. Reports suggest the origin of the *SnRK3* sub-family from green algae, and the intron-poor group first appeared in seed plants [88]. These results are consistent with phylogenetic tree studies showing that *SnRK* exon–intron numbers are highly conserved during the evolution of each sub-family. The phylogenetic tree and exon–intron structure showed that most paralogous gene pairs contain the same exon number, though some gene pairs have different exon numbers. Motif analysis revealed a close evolutionary relationship within sub-groups due to the conserved nature of motif composition among sub-families. The motif structure of each sub-family might define the biological function in which they are involved. The gene structure and sequence conservation were similar to most of the previously studied species [1,17,30,31,32,33,34,35,36].

Gene expression is regulated by external factors that are perceived through signalling mechanisms. Such signals activate specific transcription factors that, when combined with cis-acting elements in the promoter regions of genes, alter gene expression. In most genome-wide analysis studies of *SnRK* gene families, detection of *cis*-acting elements has focused on abiotic stress conditions in which the presence of hormone-, salt-, temperature-, and drought-specific *cis*-elements [1,17,30,31,32,33,34,35,36]. As the aim of our investigation was to predict the possible role of the *PvSnRK* gene family in symbiosis, we analysed symbiosis-related *cis*-elements as demonstrated in *Medicago* and *L. japonicus*. All *PvSnRK* gene sub-families contain symbiosis-related *cis*-elements. Among all *cis*-elements, ERF and MYB are the most abundant, followed by C2H2 and bHLH.

Ka/Ks analysis showed that *PvSnRK* gene family duplications either occurred slowly or are highly conserved [89]. Gene Ontology studies revealed that the *PvSnRK* genes function mostly in molecular and biological processes, specifically in cellular and metabolic processes followed by nucleotide binding activity. When we analysed expression of *PvSnRK* gene family members in different tissues, the lowest expression of any *PvSnRK* was found in aerial tissues such as young pods, flowers, seeds, and shoots. Most of the *SnRK*s were found to be expressed in root or root nodules at some stage of their development. Furthermore, transcriptomic data under early symbiotic conditions showed elevated expression levels of *PvSnRK1* s and *PvSnRK3*s, with some members being more highly induced than others. For some genes, these expression patterns were found under both root nodule and mycorrhizal symbiotic conditions, suggesting a possible role for *PvSnRK*s in the establishment of symbiotic relationships.

To predict possible interaction networks among the *PvSnRK*s and *PvSnRK*s with symbiosis-related genes, we carried out *in situ* interaction network building using the STRING database and Cytoscape. The results were interesting, as most of the PvSnRKs interact among themselves, and interaction is mediated by master regulators of cellular processes such as TOR and PKGII. Through these proteins, PvSnRKs interact with several protein phosphatases and cell cycle-regulating cyclins. We chose symbiosis-related genes based on a previously published article and identified a total of 200 genes in the *Phaseolus* genome. PvSnRK1s mostly interact with other major proteins, such as TOR and PI3Ks, which are connected to the NUPs, RAB proteins, ARPs and proteins involved in vesicle transport. On the other hand, PvSnRK2s interact with PKGII, which interact with the cell cycle regulatory proteins cyclins, CDC, and APC. In contrast, PvSNRK2s shows very few symbiotic gene interactions. Of the largest PvSnRK sub-families, PvSnRK3 interacts with NUPs, RPSs and many symbiotic genes.

Taken together, our extensive analysis of the *PvSnRK* gene family revealed structural conservation of genes across species and possible functional conservation as well. Furthermore, the principle aim of this study was to understand the putative role of *PvSnRK*s in regulating symbiotic interactions between *Phaseolus* and *Rhizobium* or *Phaseolus*; mycorrhiza are promising, as some genes contain specific *cis*-elements and showed transcript upregulation in response to symbionts. Finally, the identified *in situ* protein–protein interactions may help in predicting candidate genes for functional characterization to obtain a clear picture of the regulatory mechanisms involved.

## Figures and Tables

**Table 1:** Gene information of *Phaseolus vulgaris SnRK* gene family. *Phytozome gene ID; bp—base pairs; CDS—coding sequence; aa—amino acids; pI—isoelectric point; MW—molecular weight; kDa—kilodaltons.

Gene ID	Gene Name	Strand	*Arabidopsis * Orthologs	CDS Length, bp	Transcript Length, bp	Protein Length, aa	pI	MW, kDa	Intron Number	Integral Prediction of Protein Location	Score
**Phvul.008G039400**	*PvSnRK1.1*	Reverse	AT3G01090 (*SnRK1.1*)	1548	2155	515	8.3	58.7	9	Extracellular	2.68
**Phvul.004G032000**	*PvSnRK1.2*	Reverse		1587	2263	528	8.7	60.54	9	Extracellular	2.68
**Phvul.006G216400**	*PvSnRK2.1*	Reverse		1056	1639	351	6.1	40.25	8	Nuclear	8.5
**Phvul.002G021600**	*PvSnRK2.2*	Forward		1080	1777	359	4.7	40.77	6	Plasma membrane	5.35
**Phvul.003G247000**	*PvSnRK2.3*	Forward	AT5G66880 (*SnRK2.3*), AT3G50500 (*SnRK2.2*)	1083	1853	360	4.8	40.91	8	Plasma membrane	4.27
**Phvul.009G157800**	*PvSnRK2.4*	Forward	AT1G60940 (*SnRK2.10*), AT1G10940, (*SnRK2.4*)	1056	1596	351	5.9	40.46	8	Plasma membrane	5.7
**Phvul.003G225800**	*PvSnRK2.5*	Reverse		1017	1616	338	5.9	38.15	8	Nuclear	8.56
**Phvul.003G293600**	*PvSnRK2.6*	Reverse	AT4G33950 (SnRK2.6)	1251	1549	416	5.4	46.85	8	Nuclear	3.34
**Phvul.002G261200**	*PvSnRK2.7*	Forward	AT4G40010 (*SnRK2.7*, 84%)	1026	1712	341	5.3	38.73	8	Extracellular	2.29
**Phvul.008G176300**	*PvSnRK2.8*	Reverse	AT1G78290 (*SnRK2.8*)	1020	1316	339	5.2	38.33	8	Nuclear	3.32
**Phvul.003G012300**	*PvSnRK2.9*	Reverse		1095	1884	364	5	41.33	8	Plasma membrane	9.68
**Phvul.002G002200**	*PvSnRK2.10*	Reverse		1017	1375	338	6.7	38.65	8	Nuclear	3.32
**Phvul.002G249300**	*PvSnRK2.11*	Forward		933	1262	310	6.4	35.67	8	Nuclear	6.98
**Phvul.006G174900**	*PvSnRK3.1*	Reverse		1311	1311	436	8.8	49.77	0	Plasma membrane	9.61
**Phvul.006G076200**	*PvSnRK3.2*	Forward	AT5G58380 (*SnRK3.8*), AT5G07070 (*SnRK3.2*)	1389	1389	462	8.9	52.56	0	Plasma membrane	3,71
**Phvul.003G138278**	*PvSnRK3.3*	Forward	AT4G14580 (*SnRK3.3*), AT3G23000 (*SRK3.10*)	1320	1718	439	9.1	49.24	0	Plasma membrane	6.36
**Phvul.003G148600**	*PvSnRK3.4*	Reverse	AT5G57630 (*SnRK3.4*)	1410	1817	469	8.98	53.19	0	Plasma membrane	9.68
**Phvul.006G174800**	*PvSnRK3.5*	Forward	AT4G18700 (SnRK3.9), AT5G45810 (*SnRK3.5*)	1524	2248	507	6.6	56.8	0	Plasma membrane	5.64
**Phvul.001G248700**	*PvSnRK3.6*	Reverse		1332	2178	443	9.1	50.13	0	Extracellular	2.31
**Phvul.005G181900**	*PvSnRK3.7*	Forward		1377	2029	458	9.1	51.37	13	Plasma membrane	5.37
**Phvul.008G248900**	*PvSnRK3.8*	Reverse	AT5G07070 (*SnRK3.2*), AT5G58380 (*SnRK3.8*)	1332	1332	443	9.4	50.07	0	Plasma membrane	9.59
**Phvul.002G238800**	*PvSnRK3.9*	Forward	AT4G18700 (*SnRK3.9*), AT5G45810 (*SnRK3.5*)	1539	1830	512	7.7	57.87	0	Plasma membrane	5.4
**Phvul.007G279600**	*PvSnRK3.10*	Forward	AT3G23000 (*SnRK3.10*), AT4G14580 (*SnRK3.3*)	1269	1612	422	9.1	47.37	0	Plasma membrane	9.64
**Phvul.003G165700**	*PvSnRK3.11*	Reverse	AT5G35410 (*SnRK3.11*)	1359	2196	452	9.1	51.48	13	Plasma membrane	5.38
**Phvul.010G118300**	*PvSnRK3.12*	Forward	AT1G01140 (*SnRK3.12*)	1311	1902	436	8.8	49.6	13	Plasma membrane	5.6
**Phvul.009G145400**	*PvSnRK3.13*	Reverse	AT4G24400 (*SnRK3.13*)	1341	1790	446	6.8	50.89	13	Plasma membrane	5.88
**Phvul.003G156900**	*PvSnRK3.14*	Forward	AT4G30960 (*SnRK3.14*)	1302	2154	433	8.98	48.78	0	Plasma membrane	5.35
**Phvul.008G085000**	*PvSnRK3.15*	Forward		1158	2382	385	8.34	42.93	0	Plasma membrane	4.08
**Phvul.003G121600**	*PvSnRK3.16*	Reverse	AT3G17510 (*SnRK3.16*), AT1G48260 (*SnRK3.21*)	1266	1845	421	7.07	47.32	11	Plasma membrane	6.36
**Phvul.008G205700**	*PvSnRK3.17*	Forward	AT2G26980 (*SnRK3.17*)	1326	2122	441	6.49	50.3	13	Plasma membrane	9.54
**Phvul.006G020900**	*PvSnRK3.18*	Forward		1323	2181	440	8.7	50.11	13	Plasma membrane	9.67
**Phvul.006G076600**	*PvSnRK3.19*	Reverse	AT2G30360 (*SnRK3.22*)	1314	1899	437	6.54	48.84	1	Plasma membrane	5.55
**Phvul.009G206900**	*PvSnRK3.20*	Forward	AT4G30960 (*SnRK3.14*)	1317	2426	448	9.2	49.41	0	Plasma membrane	5.65
**Phvul.009G220200**	*PvSnRK3.21*	Reverse	AT1G48260 (*SnRK3.21*), AT3G17510 (*SnRK3.16*)	1377	1913	458	6.15	51.1	0	Plasma membrane	5.19
**Phvul.008G248800**	*PvSnRK3.22*	Forward	AT2G30360 (*SnRK3.22*)	1284	2004	427	8.28	47.68	0	Plasma membrane	5.81
**Phvul.009G213000**	*PvSnRK3.23*	Forward	AT1G30270 (*SnRK3.23*)	1374	2110	457	8.82	51.31	14	Plasma membrane	9.58
**Phvul.009G087500**	*PvSnRK3.24*	Reverse	AT5G10930 (*SnRK3.24*), AT5G25110 (*SnRK3.25*)	1341	2097	446	8.7	49.87	0	Plasma membrane	4.25
**Phvul.010G160900**	*PvSnRK3.25*	Reverse		1332	1875	443	9.1	50.54	0	Plasma membrane	9.6
**Phvul.008G084600**	*PvSnRK3.26*	Reverse		1386	1386	461	8.69	52.2	0	Plasma membrane	5.05
**Phvul.008G184400**	*PvSnRK3.27*	Reverse	AT4G24400 (*SnRK3.13*)	1341	1885	446	6.4	50.78	13	Plasma membrane	9.67
**Phvul.009G257000**	*PvSnRK3.28*	Forward		1065	1846	354	6.5	40.15	11	Plasma membrane	9.5
**Phvul.010G067600**	*PvSnRK3.29*	Forward		1404	2236	467	8.77	52.91	0	Plasma membrane	9.62

**Table 2:** One-to-one orthologous Ka/Ks relationships between *P. vulgaris* and *A. thaliana*.

Gene_ID		Gene_Name		Method	Ka	Ks	Ka_Ks	Effective Len	Average S-Sites	Average N-Sites	Divergence Time (MYA)
Locus 1 (*P. vulgaris*)	Locus 2 (*A. thaliana*)	Gene1 (*P.* vulgaris)	Gene2 (*A. thaliana*)								
Phvul.008G039400	AT3G01090	*PvSnRK1.1*	*AtSnRK1.1*	NG	0.0980	1.4703	0.0666	1536	349.1667	1186.8333	112.062
Phvul.003G247000	AT3G50500	*PvSnRK2.3*	*AtSnRK2.2*	NG	0.1337	3.0532	0.0438	1077	242.5833	834.4167	232.713
Phvul.009G157800	AT1G60940	*PvSnRK2.4*	*AtSnRK2.4*	NG	0.1045	1.5203	0.0687	1053	235.3333	817.6667	115.874
Phvul.009G157800	AT1G10940	*PvSnRK2.4*	*AtSnRK2.10*	NG	0.0979	1.8997	0.0516	1053	236.0833	816.9167	144.798
Phvul.006G076200	AT5G58380	*PvSnRK3.2*	*AtSnRK3.2*	NG	0.2279	2.1645	0.1053	1380	300.6667	1079.3333	164.974
Phvul.006G076200	AT5G07070	*PvSnRK3.2*	*AtSnRK3.8*	NG	0.2387	1.9327	0.1235	1356	290.1667	1065.8333	147.307
Phvul.003G138278	AT4G14580	*PvSnRK3.3*	*AtSnRK3.3*	NG	0.3087	1.4370	0.2148	1266	301.7500	964.2500	109.527
Phvul.003G148600	AT5G57630	*PvSnRK3.4*	*AtSnRK3.4*	NG	0.2574	2.9640	0.0869	1236	283.3333	952.6667	225.916
Phvul.006G174800	AT4G18700	*PvSnRK3.5*	*AtSnRK3.5*	NG	0.1752	2.1567	0.0812	1461	335.0000	1126.0000	164.380
Phvul.006G174800	AT5G45810	*PvSnRK3.5*	*AtSnRK3.9*	NG	0.1976	2.6915	0.0734	1428	325.6667	1102.3333	205.144
Phvul.007G279600	AT4G14580	*PvSnRK3.10*	*AtSnRK3.3*	NG	0.3474	1.9195	0.1810	1239	298.4167	940.5833	146.306
Phvul.003G165700	AT5G35410	*PvSnRK3.11*	*AtSnRK3.11*	NG	0.1988	1.9591	0.1015	1338	308.1667	1029.8333	149.325
Phvul.010G118300	AT1G01140	*PvSnRK3.12*	*AtSnRK3.12*	NG	0.1664	2.4130	0.0690	1305	287.7500	1017.2500	183.918
Phvul.009G145400	AT4G24400	*PvSnRK3.13*	*AtSnRK3.13*	NG	0.1176	1.8043	0.0652	1335	306.4167	1028.5833	137.522
Phvul.003G121600	AT3G17510	*PvSnRK3.16*	*AtSnRK3.16*	NG	0.1971	2.3211	0.0849	1263	285.8333	977.1667	176.910
Phvul.003G121600	AT1G48260	*PvSnRK3.16*	*AtSnRK3.21*	NG	0.2242	2.7543	0.0814	1263	285.2500	977.7500	209.929
Phvul.008G205700	AT2G26980	*PvSnRK3.17*	*AtSnRK3.17*	NG	0.1144	3.2890	0.0348	1320	289.8333	1030.1667	250.684
Phvul.006G076600	AT2G30360	*PvSnRK3.19*	*AtSnRK3.22*	NG	0.3027	2.8041	0.1079	1269	289.6667	979.3333	213.728
Phvul.009G220200	AT1G48260	*PvSnRK3.21*	*AtSnRK3.21*	NG	0.2411	2.1485	0.1122	1296	290.9167	1005.0833	163.756
Phvul.009G220200	AT3G17510	*PvSnRK3.21*	*AtSnRK3.16*	NG	0.2355	2.6065	0.0904	1329	296.1667	1032.8333	198.667
Phvul.008G184400	AT4G24400	*PvSnRK3.27*	*AtSnRK3.13*	NG	0.1424	1.8459	0.0771	1335	301.7500	1033.2500	140.692

**Table 3:** Putative symbiosis *cis*-acting elements of *SnRK* gene family genes in *P. vulgaris*, based on studies on symbiotic transcription factors performed in different legumes.

Motif/Transcription Factor Family	General Function	Role in Symbiosis
ARF	Transcription factors that regulate the expression of auxin response genes	Rhizobial infection [62]
B3	Play important roles in various growth and developmental processes in plants	no report
BES1	Binds to and activates Brassinosteroids target gene promoters both *in vitro* and *in vivo*	Regulate symbiotic nodulation [63]
bHLH	Controls a diverse processes from cell proliferation to cell lineage establishment	Nodule organogenesis [64]
bZIP	Plant bZIP proteins preferentially bind to DNA sequences with an ACGT core	Nodule organogenesis [65]
C2H2	The majority of such proteins characterized to date are DNA-binding transcription factors, and many have been shown to play crucial roles in the development of plants, animals and fungi	Symbiosome development [66]
Dof	Regulation of gene expression in processes such as seed storage protein synthesis in developing endosperm, light regulation of genes involved in carbohydrate metabolism, plant defense mechanisms, seed germination, gibberellin response in post-germinating aleurone, auxin response and stomata guard cell specific gene regulation	No report
ERF	Transcriptional regulation of a variety of biological processes related to growth and development, as well as various responses to environmental stimuli	Infection thread formation and nodule organogenesis [67], regulation of arbuscule branching [68]
G2-like	Regulate chloroplast development in diverse plant species	No report
HD-ZIP	Involved in developmental regulation in response to changes in environmental conditions	No report
MIKC_MADS	The best studied plant MADS-box transcription factors are those involved in floral organ identity determination	No report
MYB	The encoded proteins share the conserved MYB DNA-binding domain encoded proteins are crucial to the control of cell proliferation and differentiation in a number of cell types	Arbuscule degeneration [69], initial stages of nodulation [70]
NAC	The early reported NAC transcription factors are implicated in various aspects of plant development, response to pathogen, viral infections, and environmental stimuli	Symbiotic nodule senescence [71]
Nin-like	Nin (for nodule inception) is required for the formation of infection threads and the initiation of primordia	Infection thread initiation, nodule organogenesis, and the control of nodule number [66,72,73], regulation of recognition and signaling pathways occurring during primary steps of micorriza entrance [74]
Trihelix	Implicated in the complex transcriptional regulation of many plant genes	No report
WOX	WOX family members fulfill specialized functions in key developmental processes in plants, such as embryonic patterning, stem-cell maintenance and organ formation	Control of nodule development [75]
WRKY	WRKY proteins often act as repressors as well as activators, and that members of the family play roles in both the repression and de-repression of important plant processes	Controlling the mechanism of arbuscular mycorrhizal establishment by regulating the plant defense genes [76]
AP2	important functions in the transcriptional regulation of a variety of biological processes related to growth and development, as well as various responses to environmental stimuli	Infection thread formation and nodule organogenesis [67], regulation of arbuscule branching [68]
C3H	Embryogenesis, stress responses and hormonal pathways	No report
CAMTA	Important roles in various biological processes including disease resistance, herbivore attack response, and abiotic stress tolerance	Nodule organogenesis [77]
CPP	Play an important role in development of reproductive tissue and control of cell division in plants	No report
E2F/DP	Cell proliferation	No report
GATA	Implicated in light-responsive transcription	No report
LBD	Induction of nitrate-dependent expression of target genes in planta, cellular reprogramming processes	No report
MYB_related	Development and in stress responses	Regulating nitrogen fixation [78,79]
SBP	They play critical roles in regulating flower and fruit development as well as other physiological processes	No report
SRS	The genes are members of a small gene family of putative transcription factors in which the SHORT INTERNODES (SHI) gene is found.This suggests that SHI may act as a negative regulator of GA responses through transcriptional control.	No report
TCP	TCP genes have been found in various plant species, and new roles in plant development have been elucidated.	No report
BBR-BPC	BBR activates (GA/TC)8-containing promoters. Implicated in various aspects of plant development	No report
HSF	Heat stress transcription factors (Hsfs) are the major regulators of the plant heat stress (hs) response.	No report
FAR1	Negatively regulate flowering time under both long-day and short-day conditions	No report
RAV	Important functions in the transcriptional regulation of a variety of biological processes related to growth and development, as well as various responses to environmental stimuli.	No report
ZF-HD	Play an important role in plant growth, development and participate in responding to adversity stress	No report
TALE	Control meristem formation and/or maintenance, organ morphogenesis, organ position, and several aspects of the reproductive phase.	No report

## Data Availability

The data reported in this study are available in the supplementary information provided in the supplementary data.

## Supplementary Materials

The following supporting information can be downloaded at: https://www.mdpi.com/article/10.3390/genes13112107/s1, Figure S1: Phylogeny of the *SnRK* family genes in *Phaseolus vulgaris*. (A) Phylogenetic tree constructed with distance and neighbour-joining method using deduced full-length protein sequences of *SnRK* family genes. The phylogenetic tree was constructed using MEGA XI software with the neighbour-joining tree method with 1000 bootstrap values; Figure S2: Multiple sequence alignment of kinase domains of *Phaseolus SnRK* gene family. The alignment was generated using Clustal Omega bioinformatic program.; Figure S3: *Cis*-acting element analysis of the promoter regions of *SnRK* family genes in *Phaseolus*. Percent of various *Cis*-acting elements in 2 Kb promoter region upstream to the start codon was used to analyse the *Cis*-acting elements using Plant PAN 3.0 tool. Table S1: *Arabidopsis SnRK* gene family sequences; Table S2: *Phaseolus vulgaris SnRK* gene family information; Table S3: *PvSnRK in situ* subcellular protein localization analysis using different software; Table S4: Putative conserved motifs identified MEME in *Phaseolus* SnRK proteins. Table S5: Ka/Ks relationship of all *SnRK* gene family members of *P. vulgaris* against all *A. thaliana SnRK* members; Table S6: *PvSnRK cis*-acting elements related to rhizobial and mycorrhizal symbiosis; Table S7: PvGEA key for samples in the heat map showing the expression profiles of *SnRK* genes in various *Phaseolus* tissues.
