# Supplementary material for: Comprehensive Analysis of Phaseolus vulgaris SnRK Gene Family and Their Expression during Rhizobial and Mycorrhizal Symbiosis
Source: Genes (Basel). 2022 Nov 13;13(11):2107. doi: 10.3390/genes13112107 (PMC9691182; doi:10.3390/genes13112107)
Supplement: Supplementary file 1 [file genes-13-02107-s001.zip › Suppl. figures.pptx]

## Slide 1
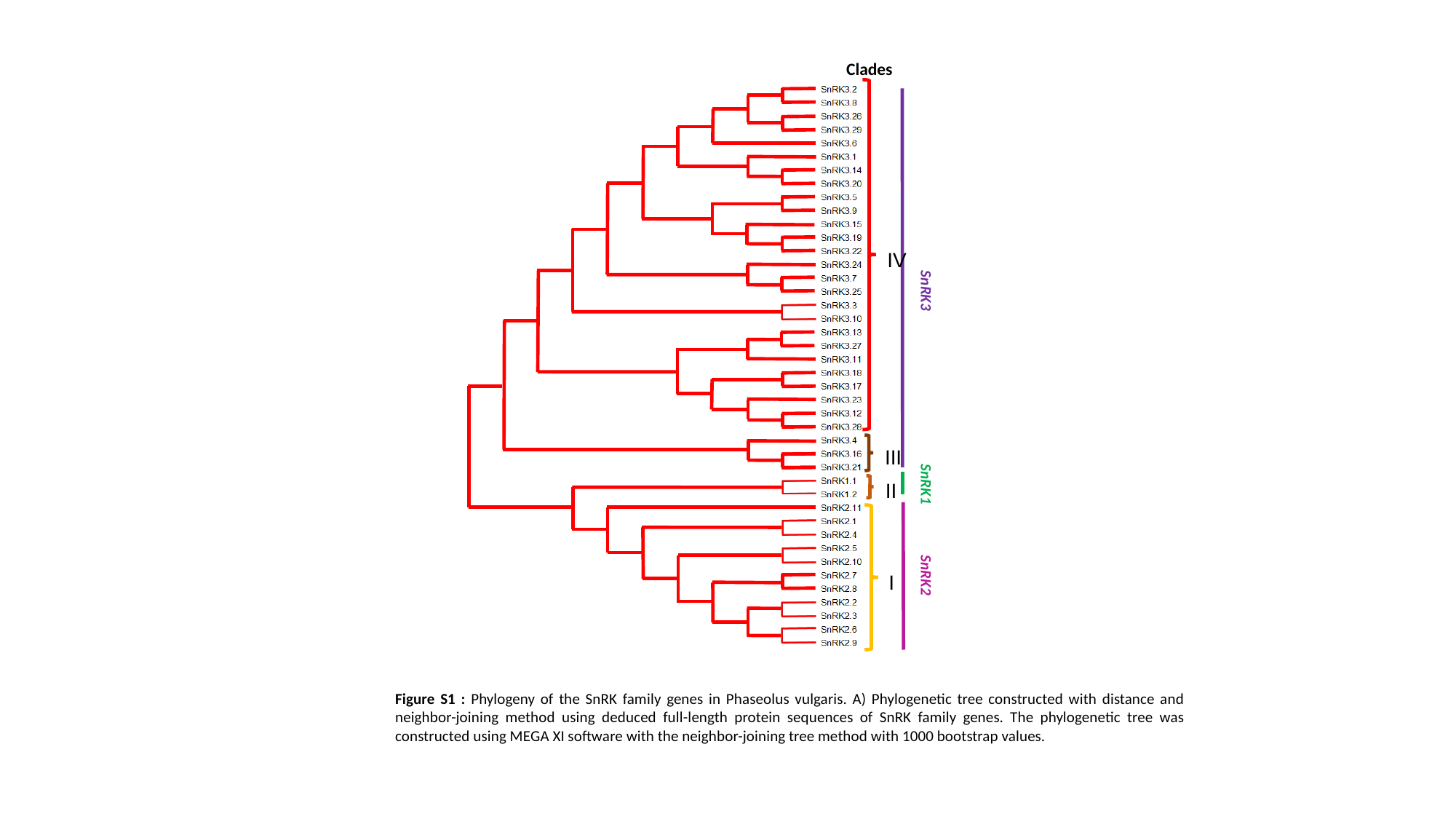

Clades
IV
SnRK3
III
SnRK1
II
SnRK2
I
Figure S1 : Phylogeny of the SnRK family genes in Phaseolus vulgaris. A) Phylogenetic tree constructed with distance and neighbor-joining method using deduced full-length protein sequences of SnRK family genes. The phylogenetic tree was constructed using MEGA XI software with the neighbor-joining tree method with 1000 bootstrap values.

## Slide 2
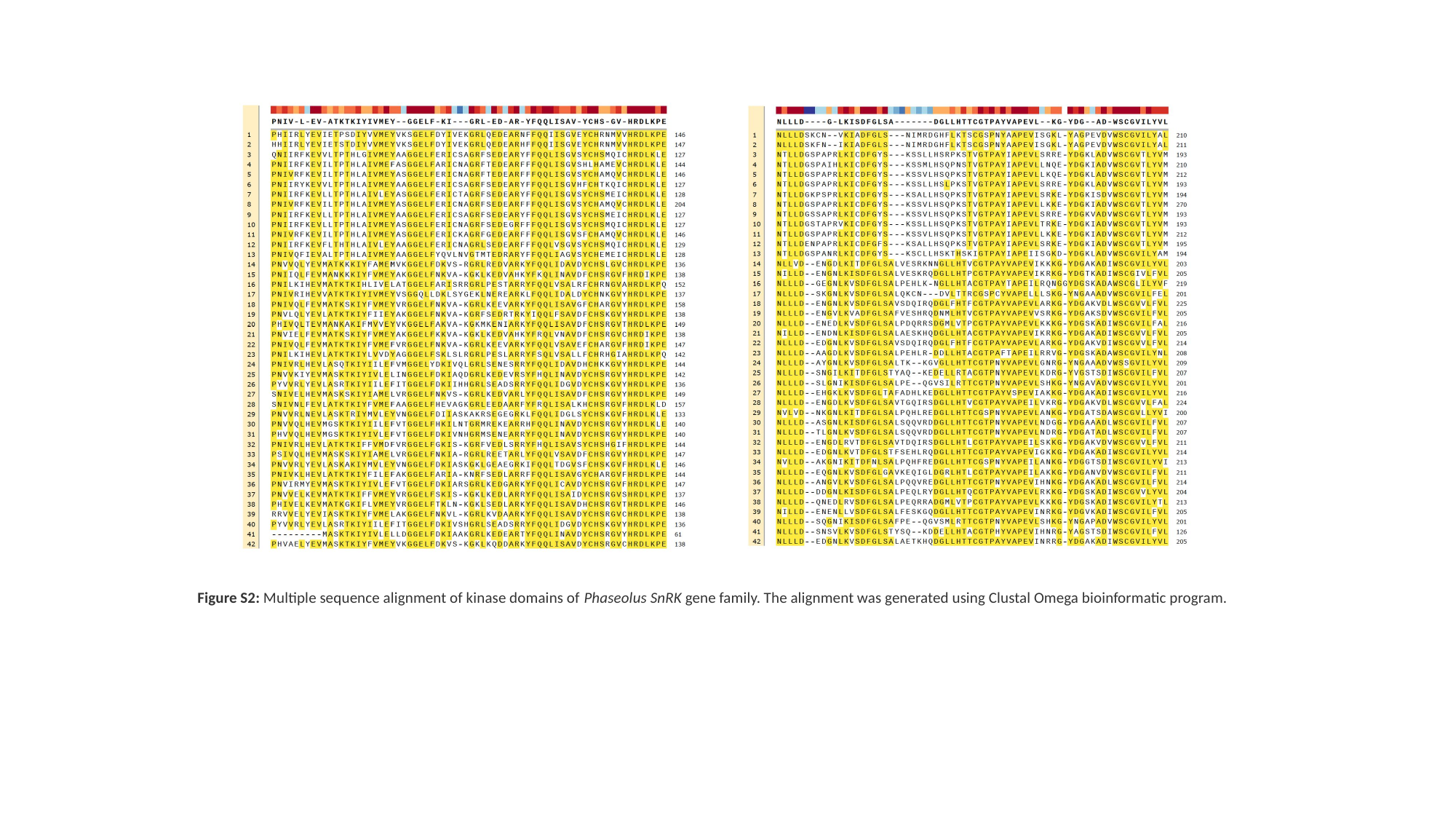

Figure S2: Multiple sequence alignment of kinase domains of Phaseolus SnRK gene family. The alignment was generated using Clustal Omega bioinformatic program.

## Slide 3
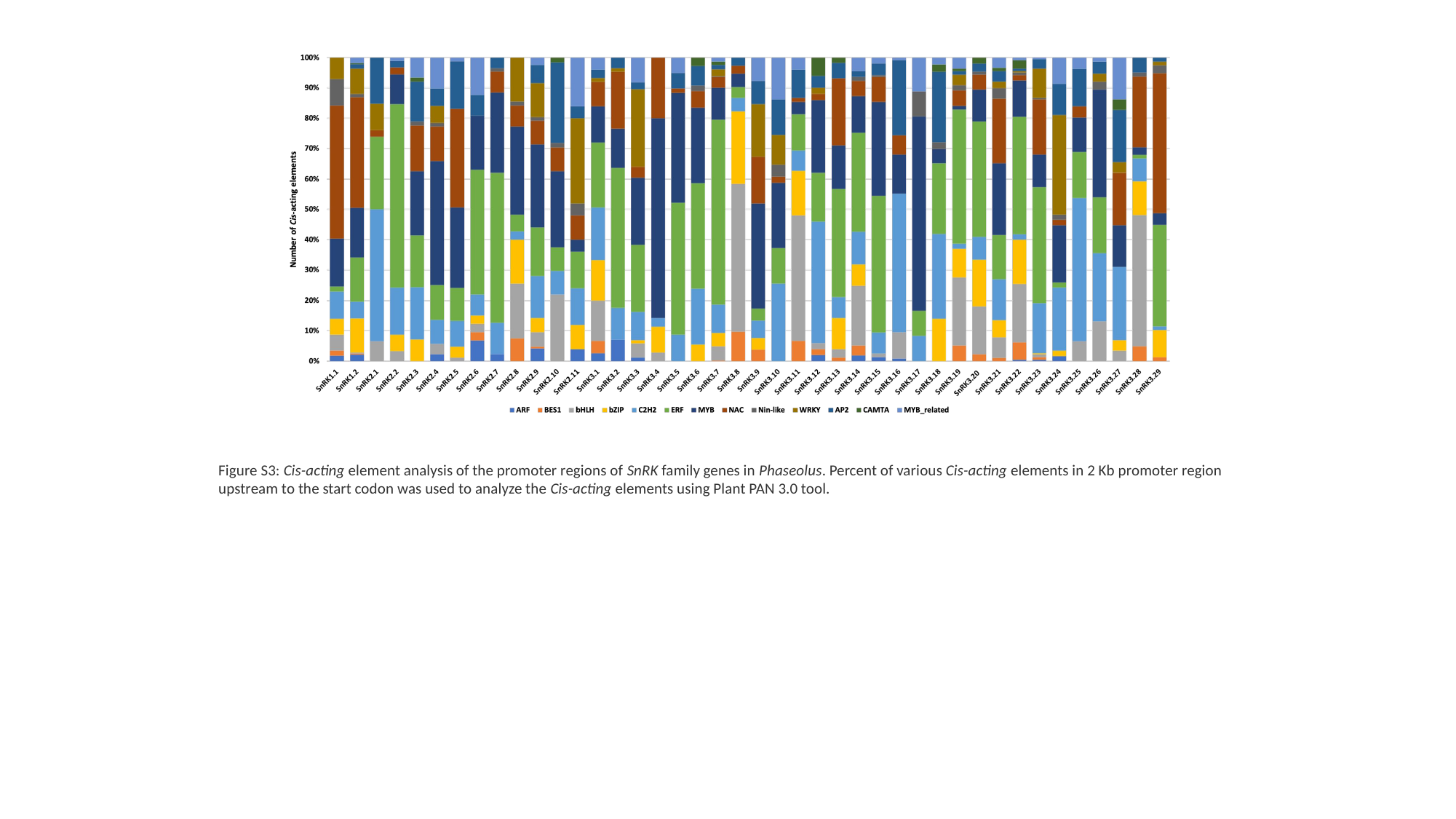

Figure S3: Cis-acting element analysis of the promoter regions of SnRK family genes in Phaseolus. Percent of various Cis-acting elements in 2 Kb promoter region upstream to the start codon was used to analyze the Cis-acting elements using Plant PAN 3.0 tool.
